# Research progress on cold tolerance key genes and mechanisms in four major northern China crops

**DOI:** 10.3389/fpls.2026.1823380

**Published:** 2026-05-21

**Authors:** Guan Liu, Hanhui Wang, Yifei Tang, Huan Gao, Yang Wang, Yan Sun, Song Yu, Dongye Zhang

**Affiliations:** 1 College of Advanced Agriculture and Ecological Environment, Heilongjiang University, Harbin, China; 2 State Key Laboratory of Tree Genetics and Breeding, College of Forestry, Northeast Forestry University, Harbin, China; 3 Department of Architecture and Urban Planning, Shandong Urban Construction Vocational College, Shandong, China

**Keywords:** cold tolerance, key genes, low-temperature stress, northern China crops, regulatory mechanism

## Abstract

Global climate change poses significant challenges to agricultural production and food security, particularly in high-latitude regions such as northern China, where low temperatures, drought, and extreme weather severely affect crop growth and development. Addressing these challenges requires a thorough understanding of crop cold tolerance, which is crucial for ensuring food security in northern China. Cold stress, including chilling and freezing injuries, could induce a range of physiological and biochemical damages in plants. Plants, however, can perceive low-temperature signals and enhance their cold tolerance through mechanisms such as the CBF/DREB transcriptional regulatory pathway. In this review, we summarize the low-temperature response and regulatory mechanisms of major crops in northern China, including wheat, maize, rice, and potato, with a focus on the unique genes and adaptive strategies these crops have evolved to improve cold tolerance. These insights not only advance our understanding of the molecular mechanisms underlying cold tolerance in key northern crops but also provide a theoretical basis for breeding cold-tolerant varieties and developing climate-resilient agriculture in northern China.

## 1 Introduction

Low temperature stress is a critical environmental constraint on plant growth and development, often causing growth retardation or even complete growth cessation, ultimately leading to substantial yield losses in major food crops (Peng et al., 2025; Roychowdhury et al., 2025). According to the injury characteristics of plants under different temperature ranges, low-temperature stress is generally classified into chilling injury (0-15°C) and freezing injury (<0°C) (Chinnusamy et al., 2007). Chilling injury occurs within the range of 0-15°C, where plant tissues do not form ice crystals, but physiological and metabolic processes become disordered, leading to organelle damage, inhibited growth and development, and disruption of multiple metabolic pathways. In contrast, freezing injury occurs below 0°C, where water inside tissues freezes to form ice crystals, causing severe cellular dehydration and structural damage, and even cell death. The severity of freezing injury is significantly greater than that of chilling injury (Guo et al., 2025; Peng et al., 2025). Consequently, freezing stress typically inflicts more severe damage than chilling stress. Many crops, such as rice (*Oryza sativa*), maize (*Zea mays*), and soybean (*Glycine max*), as well as vegetables like tomato (*Solanum lycopersicum*) and potato (*Solanum tuberosum*), are sensitive to chilling stress, whereas crops such as wheat (*Triticum aestivum*), barley (*Hordeum vulgare*), and rye (*Secale cereale*) are tolerant to freezing temperatures (Orzechowski et al., 2021; Ma et al., 2022; Elakhdar et al., 2023; Chu et al., 2024; Bukhari et al., 2025; Li et al., 2025b; Liu et al., 2025b; Zhu et al., 2025).

Plant cold tolerance is commonly assessed by observing phenotypic changes following chilling treatments, such as leaf wilting, chlorosis or yellowing, and alterations in growth and morphology. The enhancement of cold tolerance following prior exposure to low temperatures is known as cold acclimation (Zhu et al., 2007). Upon sensing low-temperature stress, plants rapidly activate a series of physiological, biochemical, and molecular responses to adapt to environmental changes and mitigate cold-induced damage (Zhou et al., 2025). Over the course of evolution, plants have developed multiple, coordinated strategies to withstand low temperatures. By integrating osmotic adjustment, maintenance of membrane stability, and regulation of stomatal aperture, plants can effectively enhance their overall cold tolerance (Steiner et al., 2020; Agurla et al., 2018; Zhang et al., 2022; Adler et al., 2025). Major staple crops in northern China, including wheat, maize, rice, and potato, are highly vulnerable to chilling and freezing stress during their growth, yet systematic studies on their responses remain limited.

Different crops exhibit significant variation in their tolerance to low-temperature stress, each with distinct physiological and adaptive characteristics. Wheat, a typical temperate crop, possesses a strong capacity for cold acclimation, whereby exposure to low temperatures in autumn progressively enhances its freezing tolerance, enabling safe overwintering (Hassan et al., 2021; Vaitkevičiūtė et al., 2024; Kosová et al., 2025). In contrast, rice, originating from tropical and subtropical regions, is a thermophilic crop that is highly sensitive to chilling stress, particularly during the seedling and reproductive stages (Li et al., 2022b; Guo et al., 2025); low temperatures could result in poor germination, seedling chlorosis, male sterility, and substantial yield loss. Similarly, maize, a thermophilic C4 plant, exhibits high sensitivity to low temperatures at the seedling stage; temperatures of 12°C can already inhibit growth, while prolonged exposure below 6°C may lead to seedling death (Ma et al., 2022; Meng et al., 2024). Potato, on the other hand, faces dual low-temperature challenges: its seedlings are highly susceptible to frost damage, and during low-temperature storage, its tubers undergo cold-induced sweetening (CIS), leading to the accumulation of reducing sugars and a marked decline in processing quality (Kou et al., 2022; Liu et al., 2025c). Given that wheat, rice, maize, and potato are major crops in northern China with large planting areas and high yield contributions, and are all affected by low-temperature stress at different developmental stages while representing typical differences between temperate and thermophilic crops in cold adaptation, they were selected for their strong representativeness and comparative value.

Therefore, we systematically searched databases including PubMed, Web of Science, and Google Scholar to collect literature on cold tolerance/freezing tolerance in four major crops published between 2018 and 2026. Priority was given to the most recent studies from the past five years, while earlier seminal works were included as foundational references. Initial screening was conducted based on titles and abstracts, with a focus on key genes related to cold tolerance in the four crops, followed by full-text review to determine the final inclusion scope. After data cleaning, VOSviewer (Perianes-Rodriguez et al., 2016) was used for visualization and analysis of research hotspots across the crops. The results indicated that current research mainly focuses on four major areas: low-temperature signaling pathways, identification of stress-resistance genes, physiological and biochemical response mechanisms, and CRISPR-based gene-editing breeding.

In contrast to previous reviews that have largely focused on a single crop or a single signaling pathway in cold tolerance, this study systematically organizes and comparatively analyzes key cold- tolerance genes in four major northern staple crops: rice, maize, wheat, and potato. We comprehensively integrate functionally validated core cold-tolerance genes from each crop and summarize their molecular characteristics across multiple levels, including low-temperature signal perception, transcriptional regulation, and metabolic response. Through this multi-crop, multi-pathway, and multi-layered systematic analysis, we not only reveal molecular differences underlying variations in cold acclimation capacity among these crops, but also facilitate the identification of core cold- tolerance gene resources, providing important references for genetic improvement of cold tolerance and for enhancing stress resilience and yield stability in northern agricultural systems.

## 2 Cold signal and physiological changes in crops

### 2.1 Low temperature signal perception and cold response signaling pathways

**Figure 1:**
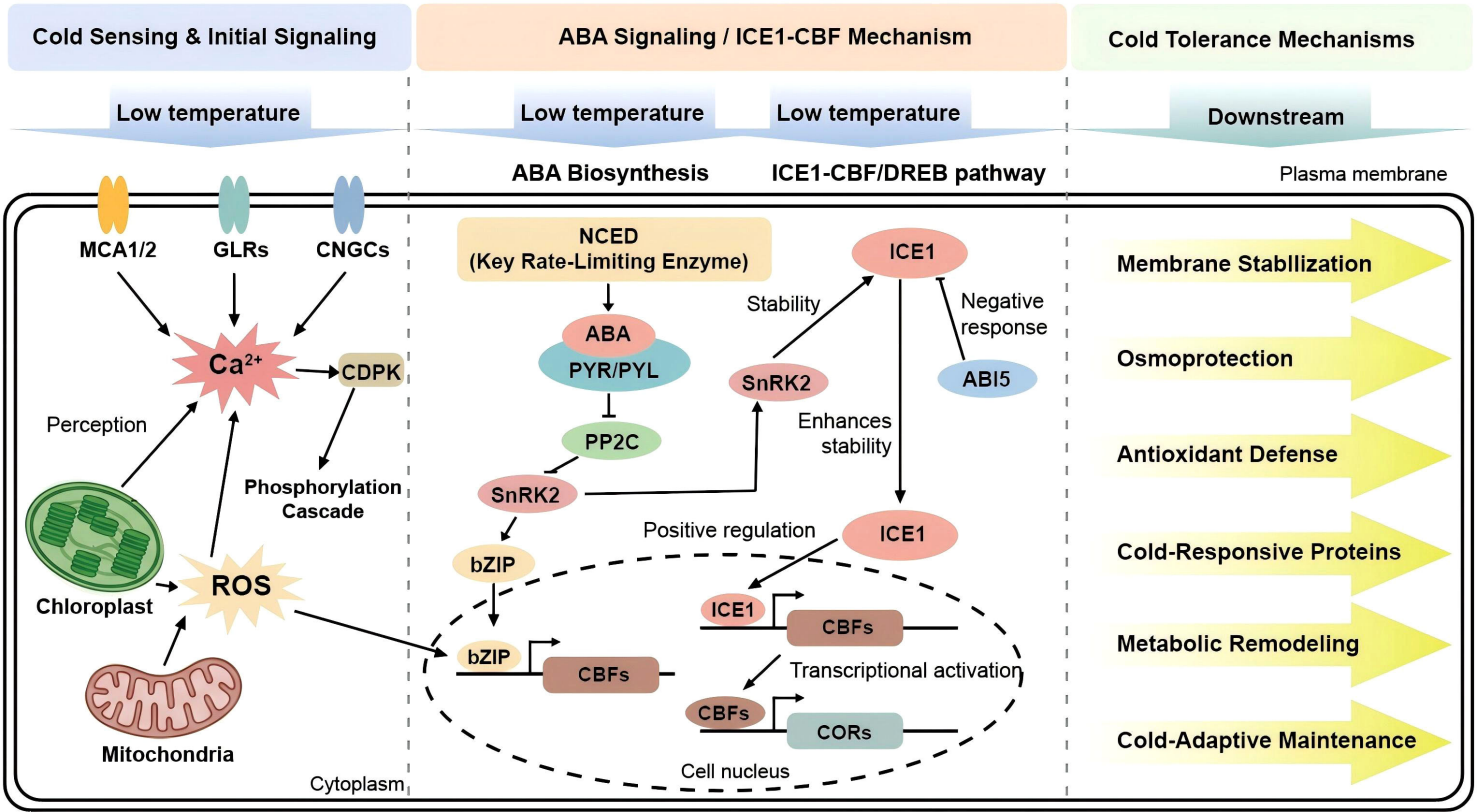
Plant cold signal perception and cold tolerance mechanism.

The main Ca^2+^ channel proteins involved in plant cold signal perception include the MCA1/MCA2 mid1-complementing activity channels, glutamate receptor-like channels (GLRs), and cyclic nucleotide-gated Ca^2+^ channels (CNGCs) (Iqbal et al., 2022). Low-temperature signals are initially perceived by the cell membrane, where changes in membrane lipid fluidity activate ion channels and receptor proteins. Organelles such as chloroplasts and mitochondria also contribute to signal perception, triggering intracellular second messengers, including Ca^2+^, reactive oxygen species (ROS), and IP_3_ (Pandey and Sanyal, 2020; Pirayesh et al., 2021). Ca^2+^ interacts with calmodulin and calcium-dependent protein kinases to initiate phosphorylation cascades, while ROS function both as signaling molecules and as components of the oxidative defense system. These signals integrate with hormone pathways to coordinately regulate key transcription factors such as ICE and CBF/DREB (Li et al., 2017; Liu et al., 2017; Tang et al., 2020). Once activated, these transcription factors enter the nucleus and bind to promoters of cold-responsive genes (e.g., *COR* genes) (Vogel et al., 2004), providing a critical molecular framework for the plant to mount comprehensive physiological responses to cold stress (**Figure 1**).

The establishment of plant cold tolerance relies on the coordinated regulation of the ABA signaling pathway and the ICE1-CBF cold response pathway (Vishwakarma et al., 2017; Gusain et al., 2023). First, ABA biosynthesis serves as the initial step in response to low temperature. Cold signals activate the key rate-limiting enzyme NCED, promoting rapid ABA accumulation; ABA is then recognized by PYR/PYL receptors, which inhibit PP2C and thereby release SnRK2 from repression (Hauser et al., 2011; Li et al., 2025a). Activated SnRK2 further phosphorylates bZIP transcription factors such as ABF, which translocate into the nucleus, bind to ABRE elements, and induce the expression of CBF and antioxidant-related genes, ultimately enhancing cold tolerance (Lin et al., 2021). In this context, ICE1 acts as a central upstream regulator of the CBF pathway by directly binding to MYC elements in the promoters of CBF/DREB1 genes, activating their expression and subsequently inducing the transcription of downstream COR genes (Tang et al., 2020; Hwarari et al., 2022). Further studies have shown that CBF/DREB expression is positively correlated with the activities of antioxidant enzymes such as SOD, CAT, and APX, and negatively correlated with oxidative damage indicators such as H_2_O_2_ and MDA (Ying et al., 2025). This relationship is explained by the ability of CBF/DREB to bind to CRT/DRE elements in the promoters of target genes, thereby activating antioxidant-related genes and enhancing ROS scavenging capacity; meanwhile, cold-induced ROS can also act as signaling molecules to promote CBF/DREB expression.

Importantly, these two pathways do not function independently but are interconnected through multiple layers of crosstalk. SnRK2 kinases in the ABA pathway, such as OST1, can directly phosphorylate ICE1, enhancing its stability and transcriptional activity, while ICE1 can in turn interact with transcription factors such as ABI5 to suppress excessive ABA responses, forming a negative feedback loop (Ding et al., 2015). In addition, factors such as ABI4 can further enhance ICE1-mediated activation of CBF (An et al., 2022). Overall, the ABA pathway and the ICE1-CBF pathway are tightly coupled through positive and negative feedback, enabling precise regulation and dynamic balance of plant cold tolerance.

### 2.2 Physiological responses and multi-omics regulation of crop cold tolerance

Plants enhance their cold tolerance through the coordinated action of multiple pathways: by adjusting membrane lipid composition to maintain the structural and functional stability of cellular membranes; by accumulating protective solutes such as proline and soluble sugars to achieve osmotic adjustment; and by strengthening antioxidant defense systems to efficiently scavenge reactive oxygen species induced by low temperatures (Satyakam et al., 2022).

In rice, osmotic regulation and antioxidant capacity are key foundations of cold tolerance. For example, in the comparison between the cold-tolerant japonica rice variety LJ25 and the cold-sensitive variety LJ11, LJ11 shows a significant increase in MDA and ROS levels under low temperature, accompanied by aggravated membrane lipid peroxidation, whereas LJ25 maintains relatively low levels of oxidative damage (Guo et al., 2022). Further analysis indicates that the activities of POD and CAT are key factors underlying their differences in cold tolerance: LJ25 significantly enhances these enzyme activities under low temperature to improve ROS scavenging capacity, while LJ11 shows a continuous decline (Guo et al., 2022). In maize, the cold-tolerant variety XY335 maintains a stable photosynthetic system and increases the levels of POD, CAT, GSH, and proline, thereby effectively reducing oxidative damage (Wang et al., 2026). Another cold-tolerant variety, MT19, further exhibits higher accumulation of soluble sugars and free proline, stronger antioxidant capacity, and lower membrane lipid peroxidation levels (Yang et al., 2026b). These results have also been validated in wheat and potato. In wheat cultivars, stronger cold tolerance is generally closely associated with higher osmotic adjustment capacity and antioxidant enzyme activities (Zhang et al., 2015). In potato seedlings under 5°C cold stress, cold-tolerant varieties similarly exhibit higher activities of POD, CAT, and SOD, along with greater accumulation of soluble sugars, soluble proteins, and proline (Ali et al., 2026). Overall, cold-tolerant germplasm across different crops commonly shows enhanced osmotic adjustment and antioxidant capacity, accompanied by lower MDA accumulation, indicating a conserved physiological adaptation pattern across species.

In this context, traditional single-omics approaches are insufficient to fully elucidate the complex mechanisms underlying low-temperature responses. The development of proteomics and metabolomics enables a more direct characterization of cold tolerance regulatory networks at the levels of protein abundance changes and metabolite accumulation, and these approaches have been widely applied in crops such as rice, maize, wheat, and potato. In rice, cold-responsive proteins are mainly involved in signal transduction, photosynthesis, and antioxidant defense (Singh and Jwa, 2013). In maize, multi-omics analyzes have revealed that photosystem stability, as well as proline and nitrogen metabolism reprogramming, play key roles in cold acclimation (Gai et al., 2025). In wheat, starch-sucrose metabolism, glycerophospholipid metabolism, and the antioxidant system represent core pathways during spike development under cold stress (Liu et al., 2025a). In potato, lipid, flavonoid, and amino acid metabolic reprogramming jointly contributes to seedling freezing tolerance (Li et al., 2025d; Yang et al., 2025). Overall, different crops share conserved pathways such as antioxidant defense, osmotic adjustment, and photosynthetic protection in response to low temperature, while also evolving species-specific regulatory modules.

## 3 Key genes involved in cold tolerance in major crops of northern China

### 3.1 Key genes involved in cold tolerance in rice

Generally, a stable mean daily temperature above 12°C is considered suitable for rice sowing, while lower temperatures could adversely affect rice growth. When low temperatures persist for more than four days, they may lead to poor germination, slow seedling growth, seedling rot, and even plant death (Li et al., 2022b). Cold tolerance in rice is a quantitative trait controlled by multiple genes and shaped by both genotype and environmental conditions. Classical genetic studies have made notable progress, for instance, identifying multiple loci associated with cold tolerance through QTL mapping (Fujino et al., 2003; Ma et al., 2015; Zhao et al., 2017; Zhang et al., 2017). At the molecular level, low temperature-induced genes can be broadly categorized into functional protein genes and regulatory protein genes, together forming the molecular network underlying rice cold tolerance (**Table 1**).

**Table 1:** Summary of key genes involved in cold stress responses in major crop species of northern China in this study.

Organisms	Factors	Functions	References
Rice	*ICE1*	Activates CBF/DREB pathway	Kanagatov et al., 2025
	*OsDREB1C*	Improves cold tolerance, nitrogen use efficiency, photosynthesis, and grain yield	Deng et al., 2024
	*OsC2H2.35*	Represses OsDREB1A/1C expression	Deng et al., 2024
	*OsCNGC14, OsCNGC16*	Plasma membrane calcium channel proteins positively regulate both heat (48 °C) and cold (6 °C) tolerance	Cui et al., 2020
	*OsCPK9*	Low-temperature–induced Ca^2+^ sensor positively regulates seedling cold toleranc	Liu et al., 2025a
	*OsLTPL159*	Lipid transfer protein enhances seedling cold tolerance by protecting chloroplasts through reducing ROS toxicity, promoting cell wall cellulose deposition, and accumulating osmoprotectants	Zhao et al., 2019a
	*CTB2*	Regulates cold tolerance throughout the entire life cycle	Li et al., 2021
	*bZIP73*	Enhances cold tolerance during both the seedling and reproductive stages in rice	Liu et al., 2019
	*OsNAC5*	Enhances rice seedling cold tolerance by activating and stabilizing OsABI5 to regulate COR genes, improving ROS scavenging, balancing ABA signaling, and alleviating membrane damage	Li et al., 2024
	*CTB6*	Maintains pollen development via ROS and lipid metabolism regulation	Cui et al., 2025
	*CTB3*	Enhances panicle cold tolerance by promoting sugar accumulation	Gao et al., 2025
	*HAN2 / OsABCB5*	Regulates auxin signaling; improves cold tolerance at seedling and booting stages	Li et al., 2025c
	*RGA4L*	Confers cold tolerance throughout the whole growth period	Gan et al., 2025
Maize	*ZmRR1*	Key positive regulator of seedling cold tolerance	Zeng et al., 2021
	*ZmbHLH30*	Positive regulator of cold tolerance at germination, bud burst, and seedling stages	Tang et al., 2026
	*ZmPgb1.1*	Overexpression enhances cold tolerance by activating the NO–BR–ZmMPK5 pathway	Mira et al., 2021
	*ZmmICE1*	Activates CBF pathway; enhances freezing tolerance	Lu et al., 2017
	*ZmASR1*	Enhances antioxidant enzyme activity under cold stress	Cai et al., 2014
	*ZmMKK1*	Activates ROS-responsive genes; participates in stress signaling	Meng and Sui, 2019
	*ZmDREB1A*	Regulates sugar synthesis and cold response	Zhao et al., 2020a; Gao et al., 2024
	*ZmMYB31*	Regulates antioxidant system and improves cold tolerance	Li et al., 2018
	*ZmMYB-IF35*	Improves cold tolerance by modulating the antioxidant system and photosynthesis	Li et al., 2019
	*COOL1*	Maintains lipid metabolic homeostasis; enhances cold tolerance at flowering stage	Zeng et al., 2025
	*bZIP68*	Negative regulator of cold tolerance; represses COOL1	Zhang et al., 2025a
	*HSF21*	Favorable allele improves cold tolerance by relieving bZIP68 repression	Zhang et al., 2025a
	*ZmDUF1645*	Heterologous expression in rice reduces seedling cold tolerance	Chen et al., 2025b
Winter wheat	*CBF*	Core regulator in cold signaling pathways	Jung et al., 2024
	*Wcor15*	Specifically induced by low temperature; serving as an important indicator for evaluating seedling cold tolerance in wheat	Takumi, 2003
	*TaSAMT1-B*	Key positive regulator of freezing tolerance	Chu et al., 2024
	*TaCYP2*	Heterologous expression in Arabidopsis increases proline content and antioxidant enzyme activities	Wang et al., 2024
	*TaMPK3*	Overexpression significantly enhances freezing tolerance by interacting with TaICE41	Han et al., 2026
	*TaICE41*	Interacts with TaMPK3; participates in cold response regulation	Han et al., 2026
*S. commersonii*	*CBF3, ICE1, HOS1, SIZ1, CDPK7, CDPK19, GOLS1*	Upregulated after cold acclimation and enhance freezing tolerance	Aversano et al., 2015
*S. tuberosum*	*StICE1*	Activates ICE–CBF–COR pathway	Wang et al., 2022b
*S. pinnatisectum*	*SpCBF1*	Overexpression elevates COR gene expression	Zhu et al., 2018
*S. commersonii & S. tuberosum*	*ScCBF1, StCBF1*	ScCBF1 has stronger effects than StCBF1 in improving cold, salt and drought tolerance	Li et al., 2022a
*S. commersonii & S. tuberosum*	*ScCBF2, StCBF2*	ScCBF2 overexpression strongly enhances freezing tolerance	Chen et al., 2025a
*S. acaule & S. tuberosum*	*SaADC1, StADC1*	Putrescine biosynthesis pathway positively regulates freezing tolerance via CBF–COR pathway and ABA signaling feedback loop	Kou et al., 2018, Kou et al., 2022
*S. tuberosum*	*StWRKY70*	Improves tolerance to cold-induced sweetening by regulating starch and sugar metabolism	Jiang et al., 2025
	*StbZIP2*	Regulates sugar metabolism via ABA signaling under cold storage	Jiang et al., 2025
	*VInv*	Controls cold-induced sweetening; knockout improves processing quality	Zhu et al., 2024

Research on rice cold tolerance has evolved from phenotypic characterization and QTL mapping to a systematic dissection of molecular regulatory networks. For example, a recent meta-QTL analysis integrating 20 cold-tolerance physiological traits and a total of 242 QTLs successfully identified 58 significantly narrowed intervals, from which 46 key cold-responsive genes were screened, covering multiple cold-related transcription factor families including WRKY, NAC, CBF/DREB, MYB, and bHLH (Kumari et al., 2024). Among these key factors, the ICE1–CBF/DREB signaling pathway has remained a central focus. The function of ICE1 is evolutionarily highly conserved; transgenic rice overexpressing ICE1 shows a 62% reduction in cold-induced damage, a 30% increase in proline content, and a 38% increase in POD activity under cold stress (Kanagatov et al., 2025), indicating significantly enhanced cold tolerance. Another study also confirmed that rice ICE1 is a functional homolog of Arabidopsis AtICE1 and plays a critical role in regulating 4°C cold responses in rice (Kanagatov et al., 2025). Moreover, overexpression of several rice *DREB1* genes has been shown to enhance stress tolerance: *OsDREB1A* improves freezing and salt tolerance in Arabidopsis; *OsDREB1F* enhances salt, drought, and cold tolerance in both Arabidopsis and rice; and *OsDREB1G* regulates low-temperature tolerance in rice (Dubouzet et al., 2003; Wang et al., 2008; Zhang et al., 2009; Moon et al., 2019). Notably, the CBF/DREB pathway in rice exhibits complex and functionally differentiated regulatory patterns under cold stress. Systematic loss-of-function mutant analyzes have demonstrated the essential roles of *OsDREB1C*, *OsDREB1E*, and *OsDREB1G* in rice low-temperature tolerance. All three single mutants showed markedly enhanced cold sensitivity, while the triple mutant *dreb1ceg* displayed far greater cold susceptibility than any individual single mutant (Wang et al., 2022a). In cold-sensitive rice, *OsDREB1C/E/G* primarily regulate basal cold tolerance rather than being involved in cold acclimation; they positively respond to low-temperature stress by regulating reactive oxygen species scavenging and cell death homeostasis, while also possessing multiple stress tolerance functions such as heat, salt, and drought tolerance (Wang et al., 2022a). Of particular interest, OsDREB1C not only enhances cold tolerance but also improves nitrogen use efficiency, photosynthetic capacity, and grain yield (Deng et al., 2024). In addition, regarding negative regulatory mechanisms downstream of the pathway, the C2H2-type zinc finger protein OsC2H2.35 can directly bind to the promoters of OsDREB1A and OsDREB1C to repress their expression, and knockout of this gene significantly enhances cold tolerance in edited lines (Deng et al., 2024).

Calcium signaling is recognized as a central regulator of plant responses to both heat and cold stress, functioning through a hierarchical cascade that proceeds sequentially from signal perception, transmission, and regulation to downstream effector responses. At the perception level, the plasma membrane-localized calcium channels OsCNGC14 and OsCNGC16, active throughout the whole growth period, perceive cold (6°C) and heat (48°C) stress and mediate cytosolic Ca^2+^ influx, generating specific calcium signal peaks; their mutants show reduced survival and increased membrane damage, confirming their key roles in stress perception and signal initiation (Cui et al., 2020). Subsequently, at the seedling stage the calcium sensor OsCPK9 is strongly induced by low temperatures and positively regulates seedling cold tolerance. Under cold stress, OsCPK9 phosphorylates and stabilizes OsSAPK8 in a Ca^2+^-dependent manner, thereby activating downstream cold responsive genes and amplifying the cold signal transduction (Liu et al., 2025b). After entering the nucleus, the signal reaches the regulation layer, where multiple transcription factors are activated. At the seedling stage, *OsNAC5* enhances cold tolerance in rice seedlings by activating and stabilizing *OsABI5*, which in turn regulates *COR* genes, improves ROS scavenging, balances ABA signaling, and reduces membrane damage, while mutants of OsNAC5 are sensitive to low temperatures (Li et al., 2024). Meanwhile, bZIP73^Jap^ functions at both the seedling stage and the reproductive stage. At the seedling stage, it forms heterodimers with bZIP71 and activates peroxidase genes to enhance cold tolerance. At the reproductive stage (binucleate stage and flowering), it regulates sugar transport genes to promote pollen sugar supply, thereby increasing seed setting rate and grain yield (Liu et al., 2019). Finally, two effector proteins directly participate in cellular protection. CTB2 (UDP-glucose sterol glucosyltransferase), active throughout the whole growth period, maintains membrane integrity and protects pollen wall structure by enhancing sterol glucosyltransferase activity, increasing seed-setting rate at the booting stage and seedling survival at the seedling stage (Li et al., 2021). The lipid transfer protein OsLTPL159, mainly at the seedling stage, protects chloroplasts by reducing reactive oxygen species toxicity, promoting cell wall cellulose deposition, and accumulating osmoprotectants; its japonica-derived allele can be used to improve cold tolerance in indica rice at the seedling stage (Zhao et al., 2019a). Together, these genes constitute a multi-layer cooperative network underlying heat and cold tolerance in rice.

In addition to the seedling stage, rice is also highly sensitive to low temperature during the reproductive stage, where cold stress at the booting and flowering stages leads to pollen abortion and reduced seed set, representing a major limitation for cultivation in northern regions (Xiao et al., 2026). In recent years, several key cold-tolerance genes have been identified, such as CTB6, which maintains pollen development by regulating ROS homeostasis and lipid metabolism in anthers; CTB3, which enhances panicle cold tolerance by promoting sugar accumulation; and HAN2/OsABCB5, which improves cold tolerance at both the seedling and booting stages through regulation of auxin signaling (Cui et al., 2025; Gao et al., 2025; Li et al., 2025c). In addition, RGA4L confers enhanced cold tolerance throughout the entire growth period (Gan et al., 2025). These findings indicate that the rice cold-response regulatory network is highly complex and exhibits pronounced stage-specific characteristics.

### 3.2 Key genes involved in cold tolerance in maize

Maize is highly sensitive to cold stress, particularly during the critical seedling stage, with an optimal growth temperature of 22-28°C. When the temperature drops below 10°C, the growth rate declines markedly, and at 6-8°C, seed germination is significantly inhibited (Zhou et al., 2022). Research on maize cold tolerance has primarily focused on the germination and seedling stages, employing linkage analysis, genome-wide association studies (GWAS), and RNA-seq to identify numerous QTLs and candidate genes (Hu et al., 2017; Ma et al., 2022). At the seedling stage, the positive regulator ZmRR1 plays a key role: its overexpression significantly reduces leaf damage and electrolyte leakage under low temperature, enhancing cold tolerance, whereas knockout mutants are cold-sensitive (Zeng et al., 2021). A 45 bp deletion in the ZmRR1 coding region gives rise to two haplotypes: the cold-tolerant HapA and the sensitive HapB. HapB is susceptible to ZmMPK8-mediated phosphorylation and degradation, whereas HapA lacks the Ser15-containing sequence, resulting in significantly higher protein stability. ZmRR1 further activates downstream cold responsive genes, including *ZmDREB1* and *ZmCesA*, thereby strengthening maize cold tolerance (Zeng et al., 2021). Another key positive regulator, *ZmbHLH30*, when overexpressed, significantly enhances cold tolerance at the germination stage, bud stage and seedling stage, with the strongest effect observed at germination (Tang et al., 2026). Transcriptome analysis indicates that differential genes in the galactose metabolism, linoleic acid metabolism, and phenylpropanoid biosynthesis pathways are likely the core mechanisms through which ZmbHLH30 regulates cold tolerance (Tang et al., 2026) (**Table 1**). Low temperatures (10°C/4°C) suppress photosynthetic rate, stomatal conductance, and transpiration, while increasing ROS accumulation and membrane lipid peroxidation. Overexpression of *ZmPgb1.1* alleviates these damages and significantly improves cold tolerance by activating the NO-BR-ZmMPK5 pathway, which inhibits ROS production by NADPH oxidases and elevates antioxidant enzyme activities, thereby protecting the integrity of the photosynthetic apparatus (Mira et al., 2021).

Beyond the above key regulators, other types of genes are also involved in maize cold tolerance regulation. For example, heterologous expression of the maize gene *ZmDUF1645* in rice, although it upregulates GIF1 to increase grain number per panicle and thousand-grain weight, suppresses starch synthesis genes, reduces antioxidant enzyme activity and proline accumulation, promotes ROS buildup and membrane lipid peroxidation, and downregulates genes involved in energy metabolism and unsaturated fatty acid biosynthesis (Chen et al., 2025b). Consequently, it weakens cold tolerance at the seedling stage in rice and simultaneously impairs rice grain appearance and eating quality. Furthermore, although ICE1 has not yet been reported in cultivated maize, ZmmICE1 was isolated from teosinte (*Zea mays ssp. mexicana*), a close wild relative of maize. As a homolog of Arabidopsis ICE1/2, ZmmICE1 exhibits similar functions by activating CBF-dependent cold responsive gene expression, thereby enhancing freezing tolerance in plants (Lu et al., 2017). Additional key genes include: *ZmASR1*, which enhances antioxidant enzyme activity; *ZmMKK1*, which activates ROS responsive genes; *ZmDREB1A*, which regulates sugar synthesis in kernels. Meanwhile, transcription factors such as *ZmMYB31* and *ZmMYB-IF35* further improve cold tolerance by modulating the antioxidant system and photosynthesis (Cai et al., 2014; Li et al., 2018, Li et al., 2019; Meng and Sui, 2019; Zhao et al., 2020a; Zhou et al., 2022).

During reproductive development in maize, low temperature can inhibit starch synthesis and the transport of photosynthates, thereby restricting grain filling and reducing kernel weight, which is an important cause of yield loss (Zhou et al., 2022). Key genes involved include DREB1A, which mediates the low-temperature response during germination by regulating ZmMIPS2; COOL1, which enhances cold tolerance at the flowering stage by maintaining lipid metabolic homeostasis and is negatively regulated by bZIP68; and favorable allelic variants of HSF21, which enhance cold tolerance at the germination and seedling stages by relieving bZIP68-mediated repression (Gao et al., 2024; Zeng et al., 2025; Zhang et al., 2025a). These studies indicate that maize cold tolerance is regulated by stage-specific functions of different genes across developmental stages, thereby forming a complex cold-response regulatory network.

### 3.3 Key genes involved in cold tolerance in wheat

Winter wheat is generally classified into cold-tolerant and cold-sensitive types. Cold-tolerant cultivars exhibit stronger adaptability to low temperatures, whereas cold-sensitive cultivars are less capable of withstanding severe cold conditions (Majláth et al., 2012). However, even cold-tolerant varieties require exposure to non-freezing low temperatures to undergo cold acclimation and thereby enhance freezing tolerance. In production, LT50 (lethal temperature for 50% mortality) is commonly used to quantify the freezing tolerance of winter wheat. Significant differences in LT50 have been observed among different genotypes. For example, the highly cold-tolerant winter wheat cultivar Norstar has an LT50 of approximately -20.7°C, whereas the less cold-tolerant cultivar Winter Manitou has an LT50 of only -14.3°C (Båga et al., 2006).

Research on wheat cold tolerance has made significant progress at the levels of transcriptomics, signaling pathways, and key genes. Transcriptome analysis revealed that the winter wheat cultivar “Dongnong Winter Wheat 1 (Dn1)” had approximately 31,000 differentially expressed genes at -10°C at the seedling stage. Low temperatures triggered activation of calcium signaling pathways, MAPK cascades, and ROS-responsive genes, while modulating transcription factors including AP2/ERF, bZIP, NAC, WRKY, bHLH, and MYB (Tian et al., 2022). Additionally, genes involved in glycolysis, starch, and sucrose metabolism were upregulated, boosting energy supply and soluble sugar accumulation to maintain cellular osmotic balance and membrane stability. While the cold-tolerant winter wheat variety “Jing 411” relies on the ABA/JA signaling pathways and proline biosynthesis pathway to confer cold tolerance under low temperature acclimation (4°C) and freezing (-5°C) conditions at the seedling stage. During acclimation, the plant accumulates soluble sugars, proline, and other compounds, activating cold-responsive genes; under freezing stress, ABA and JA levels rapidly increase, triggering emergency defense responses (Zhao et al., 2019b).

In wheat, CBF plays a crucial role in multiple low temperature signal perception pathways and in improving cold tolerance (Badawi et al., 2007; Novák et al., 2017; Jung et al., 2024), and many cold stress-related genes have been cloned and characterized. Among them, the seedling stage-specific *Wcor15* gene is specifically induced by low temperature, and its protein accumulates abundantly under cold stress, making it an important indicator for evaluating cold tolerance at the seedling stage in wheat (Takumi, 2003). Moreover, TaSAMT1-B is a key positive regulator of freezing tolerance in wheat; its expression is induced by low temperature and it catalyzes the conversion of SA to MeSA (Chu et al., 2024). Overexpression enhances freezing tolerance, whereas knockout reduces it, and it is an essential gene for BR-mediated freezing tolerance (Chu et al., 2024). Finally, through heterologous expression studies, several specific effector genes have been identified: at the seedling stage in Arabidopsis, overexpression of TaCYP2 increases survival, reduces membrane damage and ROS accumulation, and elevates proline content and antioxidant enzyme activities. While overexpression of TaMPK3 significantly enhances freezing tolerance through interaction with *TaICE41* (Wang et al., 2024; Han et al., 2026).

Wheat cold tolerance research has also focused on the reproductive stage, particularly spring low-temperature stress during the booting and meiotic phases. Transcriptome analyzes have shown that differentially expressed genes under cold treatment are mainly enriched in pathways related to hormone signal transduction, carbohydrate metabolism, and pollen wall development. The cold-tolerant cultivar Chuanmai 104 (CM104) exhibits stronger osmotic adjustment capacity, higher antioxidant enzyme activities, and more complete pollen development under low temperature at the booting stage, thereby maintaining a relatively high seed set (Liu et al., 2025a). In contrast, the cold-sensitive cultivar CM42 shows severe pollen abortion under the same conditions. Multi-omics analyzes further indicate that starch and sucrose metabolism, as well as glycerophospholipid metabolism, are core pathways underlying cold tolerance during wheat booting. Meanwhile, QTL mapping and GWAS studies are being used to identify key genetic loci associated with reproductive-stage frost tolerance in wheat, providing valuable targets for molecular breeding (Zhao et al., 2020b; Bolouri et al., 2023; Zhang et al., 2023).

### 3.4 Key genes involved in cold tolerance in potato

Different potato varieties exhibit significant differences in cold tolerance. At the seedling stage, common cultivated varieties have a semi-lethal temperature of approximately -2°C to -2.5°C both before and after cold acclimation, whereas wild species display varying levels of inherent cold tolerance and acclimation capacity, with most wild species showing much higher frost resistance than cultivated ones (Chen and L.P., 1980). Through the evaluation of cold tolerance at the seedling stage in 40 potato accessions, and using *S. piurana* and *S. chomatophilum* as parents, a set of germplasm with enhanced cold tolerance was successfully developed. Meanwhile, the freezing tolerance gene from the cold-hardy wild potato *Solanum malmeanum* was introduced into the cold-sensitive cultivated potato *S. tuberosum*, resulting in 51 somatic hybrids (fusion rate 63.75%), mainly hexaploid and octoploid, with freezing tolerance intermediate between the parents and markedly improved cold acclimation compared to the cultivated parent (Tu et al., 2021).

Among wild species, the diploid wild *S. commersonii* exhibits strong cold tolerance. After cold acclimation, transcriptome sequencing revealed the upregulation of 855 genes, including CBF3, ICE1, HOS1 (highly expressed osmotically responsive gene 1), SIZ1 (SUMO E3 ligase), calcium-dependent protein kinases (such as CDPK7, CDPK19), and galactinol synthase (GOLS1), all of which enhance freezing tolerance (Aversano et al., 2015). StICE1, a MYC-type bHLH transcription factor localized in the nucleus, functions mainly through post-translational modifications under low temperature. It enhances cold tolerance by both activating the ICE-CBF-COR pathway (upregulating CBF2/CBF3 and COR genes) and directly regulating StLTI6A to maintain membrane stability, thereby reducing oxidative damage and improving plant adaptation to cold stress (Wang et al., 2022b).

In potato, the CBF gene family is organized into two clusters: CBF3-CBF1-CBF2-CBF2B and CBF5-CBF4. Comparative analysis of the CBF gene family between Solanum tuberosum and Solanum commersonii revealed that CBF4 and CBF5 are present and linked in *S. tuberosum*, whereas only CBF4 is detected in *S. commersonii* (Pennycooke et al., 2008). In plants overexpressing *SpCBF1*, the transcript levels of cold-regulated (COR) genes were significantly elevated, and physiological indicators related to cold tolerance, such as SOD, soluble protein, MDA, proline, and soluble sugars, were all higher than in wild-type plants (Zhu et al., 2018), indicating that *SpCBF1* overexpression enhances cold tolerance in potato. Studies have also reported that *ScCBF1* from *S. commersonii* exhibits significantly greater effectiveness than StCBF1 from *S. tuberosum* in enhancing potato tolerance to cold, salt, and drought stresses (Li et al., 2022a). Analysis of CBF2 homologs across 46 potato genotypes revealed that a key residue, site A, in the activation domain is closely associated with freezing tolerance (Chen et al., 2025a). In the cold-tolerant wild species *So. commersonii* (*ScCBF2*), this residue is a serine-proline type, whereas in the sensitive cultivated species *S. tuberosum* (*StCBF2*), it is either deleted or replaced by leucine. Overexpression of *ScCBF2* markedly enhances both basal and cold-acclimation-induced freezing tolerance in potato without affecting tuber yield, whereas *StCBF2* shows only limited effects (Chen et al., 2025a). These findings indicate that structural variation in CBF2, through differential regulation of GolS3-mediated raffinose biosynthesis, is a key determinant of freezing tolerance differences in potato.

In addition, studies have shown that the arginine decarboxylase gene *ADC1*-mediated putrescine biosynthesis pathway plays a key role in cold-acclimation-induced freezing tolerance in potato. The cold-tolerant wild species *S. acaule* (Aca) specifically upregulates *ADC1* expression and accumulates putrescine under low temperature, whereas the sensitive cultivated species *S. tuberosum* (Tub) lacks this feature. Exogenous application of putrescine or overexpression of Aca’s *SaADC1* gene in the sensitive variety E3 significantly enhances freezing tolerance after cold acclimation, acting through the activation of CBF genes (CBF1-4) and downstream COR genes (e.g., *DHN10*, *RD17*) (Kou et al., 2018). Meanwhile, transgenic potatoes overexpressing *ADC1* can significantly activate the ABA signaling pathway under low-temperature treatment, leading to upregulation of PYL receptors, SnRK2 kinases, and the transcription factor AREB2. Exogenous application of ABA not only reduces electrolyte leakage and enhances freezing tolerance under low temperature, but also promotes ADC1 promoter activity through AREB/ABF/ABI5 family transcription factors (especially AREB2), further stimulating putrescine accumulation (Kou et al., 2022). This establishes a positive feedback loop of “putrescine activating ABA signaling-ABA reciprocally enhancing putrescine biosynthesis,” which synergistically strengthens cold-acclimation-induced freezing tolerance in potato.

Research on potato low-temperature responses, in addition to frost stress at the seedling stage, has also focused on two key processes: cold-induced sweetening during tuber storage and dormancy regulation. During cold storage, tubers readily undergo “cold-induced sweetening” at 4°C, which leads to browning and the formation of acrylamide during processing (Liu et al., 2025c). Related studies have shown that *StWRKY70* can enhance tolerance to cold-induced sweetening by coordinating starch degradation and sugar metabolism, while *StbZIP2* regulates the expression of sugar metabolism-related genes through the ABA signaling pathway (Jiang et al., 2025). In addition, CRISPR/Cas9-mediated knockout of *VInv* has been applied in multiple cultivars and can significantly improve processing quality under low-temperature storage conditions (Zhu et al., 2024). In terms of tuber dormancy regulation, low temperature and other environmental factors control the transition between dormancy and sprouting by modulating the balance between ABA and GA (Dogramaci et al., 2026). These studies provide an important foundation for improving potato quality and production efficiency through molecular regulation and optimized storage conditions.

## 4 Gene editing strategies for different crops

CRISPR/Cas9 gene editing technology provides an important tool for the precise improvement of crop cold tolerance. Compared with conventional breeding methods, it offers advantages such as simplicity of operation, high editing precision, short breeding cycle, and the ability to achieve multi-gene coordinated regulation, enabling the rapid generation of new germplasm with enhanced cold tolerance through gene knockout, replacement, or site-directed mutagenesis (Chen et al., 2024; Uranga et al., 2024). In recent years, this technology has been applied in major northern crops such as rice, maize, wheat, and potato (**Table 2**); however, due to differences in genome structure and genetic characteristics, the specific editing strategies vary among species.

**Table 2:** Representative applications of CRISPR/Cas9 gene editing technology for improving cold tolerance in major crops.

Organisms	Gene	Function	References
Rice	*OsCS511*	Knockout increases survival rate and chlorophyll content under 18 °C cold stress, enhancing cold tolerance	Park et al., 2024
	*OsPIN5b/GS3/OsMYB30*	Knockout improves panicle length, grain size, yield, and cold tolerance; overcomes yield–stress trade-off	Zeng et al., 2020
	*pri-miR1850/NPR3*	pri-miR1850 knockout enhances cold tolerance, NPR3 knockout reduces tolerance	Shen et al., 2025
Maize	*ZmICE1*	Knockout increases cold sensitivity; affects amino acid metabolism and ROS accumulation	Jiang et al., 2022
	*ZmbHLH30*	Overexpression enhances cold tolerance; knockout reduces tolerance at germination and seedling stages	Tang et al., 2026
Wheat	*TaWRKY115*	Knockout reduces cold tolerance via TaMYB4–CBFs pathway	Chu et al., 2024
	*TaTPS11-6D*	Knockout decreases cold tolerance; involved in sugar metabolism and hormone regulation	Jinyuan Liu et al., 2026
	*TaSAMT1*	Knockout reduces cold tolerance; integrates BR and SA signaling and epigenetic regulation	Chu et al., 2024
Potato	*ScF3’H*	Regulates flavonoid metabolism; knockout reveals its positive role in cold tolerance	Bao et al., 2025
	*VInv/InvVac*	Knockout reduces reducing sugar accumulation during cold storage; improves tuber quality	Yasmeen et al., 2022; Egorova et al., 2024

Rice is one of the crops in which genome editing has been most extensively applied. In single-gene functional validation, the seedling-stage cold-sensitive candidate gene *OsCS511*, previously associated through QTL mapping, was functionally analyzed using CRISPR Cas9-mediated knockout (Park et al., 2024). No significant differences between edited lines and wild type under normal growth conditions, whereas under 18°C cold stress, the edited lines exhibited higher survival rates and chlorophyll content, indicating enhanced cold tolerance. In terms of multi-gene coordinated editing, three negative regulatory genes, *OsPIN5b*, *GS3*, and *OsMYB30*, which independently regulate panicle architecture, grain size, and low-temperature tolerance in rice, were simultaneously edited (Zeng et al., 2020). A total of eight triple-mutant lines were obtained, among which two T2 lines showed increased panicle length, larger grain size, and higher yield, as well as significantly improved survival rates under 4°C cold stress, thereby overcoming the traditional trade-off between yield and stress resistance (Zeng et al., 2020). Furthermore, in the analysis of non-coding RNA regulatory networks, a rice-specific cold-responsive miR1850 was identified through miRNA microarray screening under cold stress, and gene editing mutants of both pri-miR1850 and its target gene *NPR3* were generated (Shen et al., 2025). Disruption of pri-miR1850 increased rice survival at 4°C, whereas knockout of *NPR3* reduced cold tolerance, suggesting that miR1850.1 regulates rice cold response at the seedling and booting stages by targeting *NPR3* (Shen et al., 2025).

In maize cold-tolerance breeding, the current core strategy mainly relies on precise modification of single key genes to achieve trait improvement. Significant progress has been made in single gene editing of key regulatory factors such as *ZmbHLH30* and *ZmICE1*. For example, under 4°C cold stress, knockout of *ZmICE1* significantly increases plant cold sensitivity, exacerbates leaf damage, and is accompanied by disrupted amino acid metabolism and excessive accumulation of mitochondrial ROS (Jiang et al., 2022). In contrast, *ZmbHLH30* overexpression lines show markedly enhanced cold tolerance at the germination, bud, and seedling stages, with a 0.366 increase in cold tolerance difference at the germination stage compared with the control, while knockout mutants decrease by 0.399 (Tang et al., 2026). Although direct cases of simultaneous editing of multiple cold-tolerance-related genes remain limited, the emergence of new genome editing tools such as CRISPR Cas12a provides new technological possibilities for high-throughput and multi-target genome editing in maize (Gong et al., 2021; Liu et al., 2024; Namata et al., 2025).

Wheat is an allohexaploid species containing a large number of homologous genes across the A, B, and D subgenomes. Due to this genetic redundancy, single-gene knockouts are often compensated by other homologs, making it difficult to produce clear phenotypic effects and thereby increasing the difficulty of cold-tolerance gene editing. Nevertheless, in recent years, CRISPR Cas9 technology has been successfully applied to the functional validation and improvement of multiple wheat cold-tolerance genes. For example, knockout of *TaWRKY115* reduces cold tolerance, which is associated with regulation of the TaMYB4-CBFs pathway; knockout of *TaTPS11-6D* also decreases cold tolerance, involving sugar metabolism and hormone regulation; and knockout of *TaSAMT1* leads to reduced cold tolerance, involving the integration of brassinosteroid and salicylic acid signaling as well as epigenetic regulation (Chu et al., 2024; Jinyuan Liu et al., 2026). These studies demonstrate that CRISPR Cas9 has strong potential for improving cold tolerance in wheat.

Potato is a tetraploid crop, and compared with wheat, research on cold-tolerance gene editing in potato started later. In addition, its long-term reliance on vegetative propagation and relatively low transformation efficiency have limited progress in improving freezing tolerance. In studies on freezing tolerance, CRISPR/Cas9-mediated loss-of-function mutants of *ScF3’H* revealed that this gene enhances low-temperature tolerance in the wild species *S. commersonii* by regulating flavonoid metabolism (Bao et al., 2025). For improving tuber cold storage quality, knockout of *VInv* and *InvVac* significantly reduces the accumulation of reducing sugars during low-temperature storage (Yasmeen et al., 2022; Egorova et al., 2024). These findings demonstrate that gene editing technologies have promising practical applications in potato.

## 5 Conclusion and future expectations

Different crops have evolved both specific and conserved mechanisms for cold adaptation. In rice, cold tolerance is established through *OsCNGC14/16*-mediated calcium signaling, the *bZIP73^JAP^*-regulated whole-life-stage cold response pathway, and *CTB2*-maintained membrane integrity. In maize, cold tolerance relies on *ZmRR1*-mediated protein stability, *ZmbHLH30*-driven metabolic remodeling, and *ZmPgb1.1* protecting photosynthetic tissues via the NO-BR signaling pathway. In wheat, cold acclimation enhances freezing tolerance by activating the CBF family, the *TaSAMT1*-mediated hormone network, and the *TaMPK3-TaICE41* interaction. In potato, superior alleles from wild species (e.g., *ScCBF2*, *SaADC1*) improve cold tolerance through the CBF2-GolS3 raffinose biosynthesis pathway and the ADC1-ABA positive feedback loop (**Table 1**). Current research has mainly focused on the overexpression or knockout analysis of single genes, with relatively limited attention to epistatic effects among multiple genes and their functional variation in different genetic backgrounds. The synergistic and antagonistic relationships among multiple genes and pathways remain a relatively weakly understood aspect, making it difficult to predict the actual phenotypes after pyramiding multiple “favorable” alleles. Therefore, although we have summarized representative cold-tolerance genes in these four major crops (**Table 1**), it should be recognized that this knowledge is still relatively fragmented and remains some distance away from guiding multi-gene pyramiding breeding. Moreover, it should be noted that this review focuses on four staple crops, while soybean, sorghum, and foxtail millet, which are also important northern crops and similarly face low temperature stress, also have relatively limited exploration of cold-tolerance genetic resources, and thus deserve to be included in future comparative research frameworks.

Currently, advanced phenomics technologies, such as three-dimensional phenotyping, micro-CT, and super-resolution microscopy (Yang et al., 2026a), provide powerful tools for precisely quantifying cold stress-related traits at micro and dynamic scales, including membrane integrity, chlorophyll fluorescence, and ROS distribution. Combined with spatial transcriptomics and single cell sequencing, these approaches can reveal, at unprecedented resolution, the spatiotemporal specificity and interaction networks of gene expression in different tissues and cell types under low temperature, as well as the heterogeneous responses of complex tissues such as meristems and vascular bundles (Luo et al., 2024; Zhang et al., 2025b). Meanwhile, CRISPR/Cas9 technology, known for its efficiency and precision, has become a key tool for simultaneously manipulating multiple cold-related genes (Mansi and Danai, 2026), enhancing protective protein accumulation, and creating new germplasm with stacked superior cold-tolerant alleles. However, effectively translating these technological advances into breeding applications still requires overcoming the transition from description to prediction and from single-factor to multi-factor approaches. Future research should place greater emphasis on integrating different technologies and on bridging the gap between laboratory conditions and real field environments.

Although significant progress has been made in cold tolerance research in northern crops, further efforts are still required under the context of accelerating global climate change. First, system biology approaches should be employed to integrate signal perception, transcriptional regulation, and metabolic reprogramming, thereby constructing a more comprehensive regulatory network for cold tolerance. Second, greater attention should be given to the discovery and functional validation of cold-tolerance genes from wild germplasm, as well as strategies for their efficient introgression into cultivated species while minimizing the introduction of undesirable traits. Third, research should be extended from controlled laboratory conditions to real field environments, with systematic evaluation of crop cold tolerance across multiple locations, years, and combined stress conditions, while emphasizing the dynamic processes of cold acclimation, deacclimation, and reacclimation. Fourth, an integrated “gene-phenotype-environment” evaluation system should be established to promote data sharing and standardization, enabling more rapid translation of laboratory findings into breeding applications and facilitating the development of cold-tolerant, high-yielding crop varieties adapted to northern climates.
